# Laparoscopic Sleeve Gastrectomy (LSG) in a Patient With Situs Inversus Totalis (SIT): A Case Report

**DOI:** 10.7759/cureus.40873

**Published:** 2023-06-23

**Authors:** Héctor J Pérez Corzo, José S Verboonen Sotelo, Jeffry Romero Manzano, Roberto E Salgado Salas, Isaac Esparza

**Affiliations:** 1 Bariatric Surgery, Renew Bariatrics, Tijuana, MEX; 2 Bariatric Surgery, Obesity Goodbye Center, Tijuana, MEX

**Keywords:** bariatric & metabolic surgery fbms, obesity, bariatric surgery, situs inversus, laparoscopic sleeve gastrectomy

## Abstract

Obesity is associated with several preventable health issues, such as diabetes mellitus and hypertension. Bariatric surgery has shown potential in treating obesity. Laparoscopic sleeve gastrectomy (LSG) is one of several bariatric surgical techniques gaining popularity as a primary procedure. Situs inversus totalis (SIT) is an uncommon hereditary abnormality that can present challenges in laparoscopic surgery due to the mirror-image anatomy. We present the case of a 54-year-old female with a body mass index (BMI) of 54.36 kg/m^2^. She was diagnosed with SIT and had no other known diseases, medication use, or allergies. We performed a conventional LSG, modifying the original trocar port positions to match the anatomy. LSG is a safe and effective procedure for patients with SIT. Preoperative diagnosis can help reduce the risk of complications by facilitating proper surgical planning.

## Introduction

Obesity has emerged as a significant public health problem [1]. It is associated with various preventable health issues, including diabetes mellitus and hypertension [2]. Bariatric surgery has shown promise in treating obesity. Laparoscopic sleeve gastrectomy (LSG) is one of several bariatric surgical techniques gaining popularity as a primary intervention. Furthermore, these treatments have demonstrated preventive benefits against metabolic syndromes, such as diabetes mellitus, cancer incidence, and sometimes even improved survival rates [3-4].

Situs inversus totalis (SIT) is a rare hereditary abnormality that can pose challenges for laparoscopic surgery due to the mirror-image anatomy [4]. SIT is an autosomal recessive disease that affects a small percentage of the population (0.005%-0.02%). It involves complete intra-abdominal organ transposition. Most individuals with SIT do not experience symptoms or consequences as the interaction between the organs remains unaltered [5].

## Case presentation

We present the case of a 54-year-old female who underwent gastric sleeve surgery. The patient had a height of 1.57 m, weighed 134 kg, and had a BMI of 54.36. She had a surgical history of cholecystectomy during adolescence. Additionally, she was diagnosed with SIT, with no other known medical conditions, current medication use, or allergies. Despite following multiple exercise and diet regimens, as well as using Phentermine, she had been unsuccessful in achieving weight loss. Given her prior diagnosis of SIT, our team was prepared to address this unique clinical situation. Preoperative and laboratory evaluations were within the expected normal range. A chest X-ray revealed dextrocardia.

**Figure 1:**
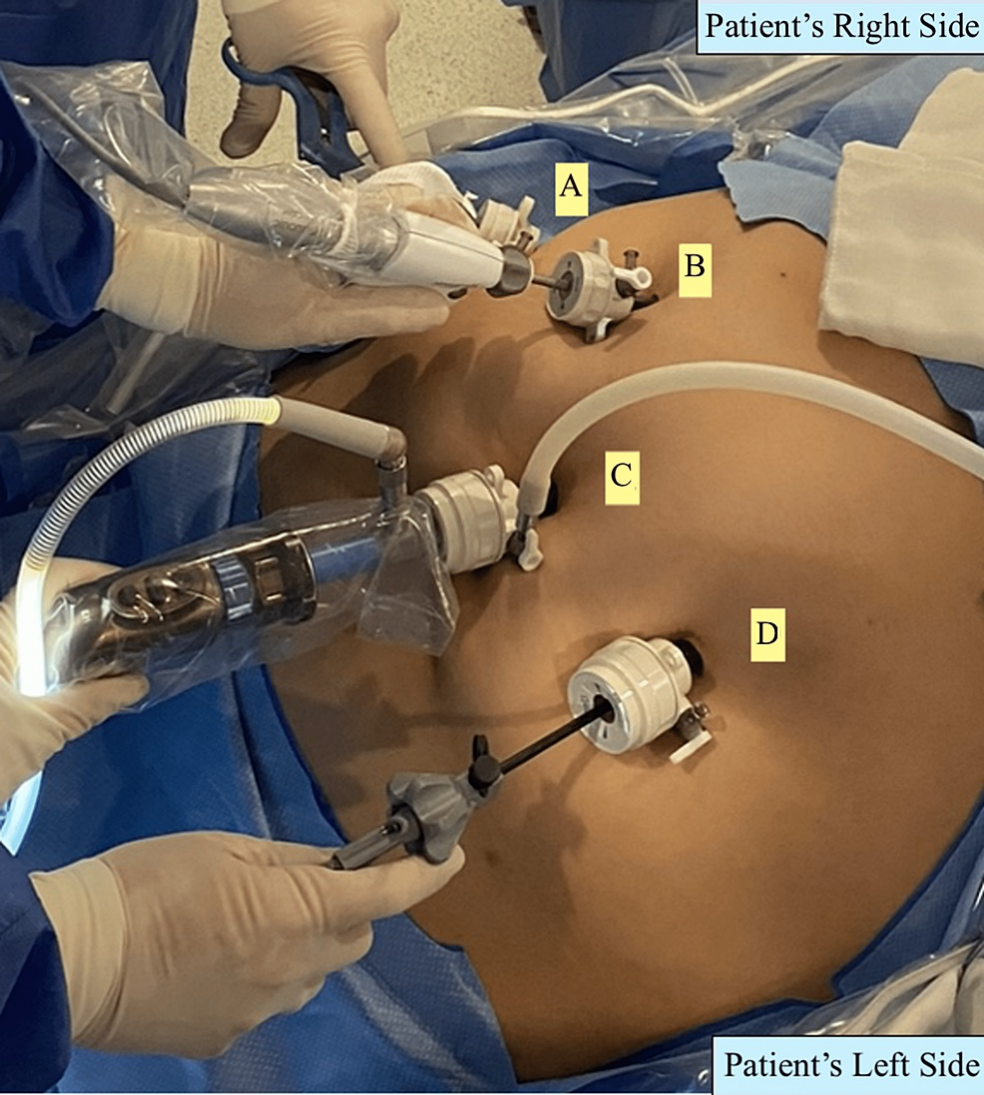
Modified trocar ports. Supraumbilical right anterior axillary line (A), supraumbilical right midclavicular line (B), supraumbilical midline (C), and supraumbilical left midclavicular line (D).

**Figure 2:**
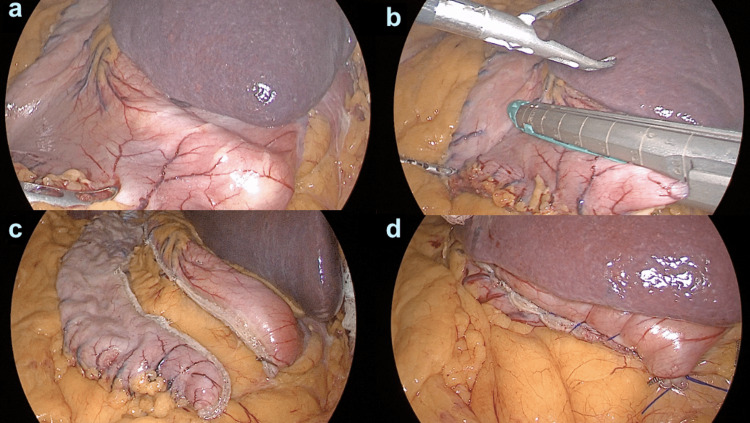
Laparoscopic gastric sleeve procedure. a: First laparoscopic view. b: Stomach stapling. c: New gastric pouch. d: Reinforced staple line

We performed a conventional laparoscopic gastric sleeve, with the surgeon positioned on the right side of the patient and the assistant on the left side, the patient is placed in dorsal decubitus position with the French technique and under general anesthesia. Using a Veress needle, insufflation is performed at the right subcostal border along the mid-clavicular line, achieving pneumoperitoneum. A 12-mm trocar is then placed supraumbilically along the midline, and a laparoscopic lens is inserted for direct visualization. Additional trocars are inserted under direct vision, including a 12-mm trocar supraumbilically along the right mid-clavicular line, a 5-mm trocar at the same level along the right anterior axillary line, and a 12-mm trocar supraumbilically along the left mid-clavicular line (Figure 1). A 36 Fr orogastric tube was used as a guide during the gastric sleeve procedure (Figure 2). A Feng linear stapler was used to perform five firings of 4.8-mm staples (green color). The staple line was reinforced with Prolene 2-0 to ensure strength and stability as we regularly do, and an intraoperative pneumatic test was performed to ensure no leaks. The surgery had minimal bleeding (10 mL), a surgical time of 45 min, and a Penrose drainage tube was placed. The patient had a favorable prognosis.

 

After the surgery, the patient's recovery was uneventful, as expected. We implemented a standard analgesic and antiemetic regimen, consisting of ketorolac/paracetamol and ondansetron, respectively. The patient was able to resume oral intake with a liquid diet approximately 5 h post-surgery and started walking without any issues 6 h after the procedure. A follow-up fluoroscopy conducted at the 24-h mark revealed no abnormalities, and the drainage tube was removed, resulting in a serohematic output of 20 mL. The patient was discharged 48 h post-surgery, and no complications were observed. Six months after surgery, the patient has lost 50 kg, which is equivalent to approximately 60% of excess weight loss.

## Discussion

This case report describes a patient who underwent gastric sleeve surgery with a history of SIT previously diagnosed. SIT is a rare condition. Therefore, surgeons treating many patients should expect to see it once or twice throughout their careers [6]. Since bariatric surgery is one of the most effective treatments for obesity, it is anticipated that more patients will opt for it in the future. As a result, more patients may present with SIT, either as a preexisting condition or as an unexpected finding during the preoperative evaluation. In these individuals, LSG surgery is a safe and effective technique [7].

Few cases of situs inversus in bariatric surgery have been reported over the years [6]. It is essential to identify if our patients have these kinds of anatomical alterations. Although SIT does not represent a severe health condition, proper surgical planning can help reduce the risk of complications [8]. There are no guidelines that provide guidance on the optimal approach for these patients. Therefore, the surgeon and surgical team must be capable of adapting conventional techniques, as we did in our case, to better approach the patient's abdominal cavity.

The average surgical time for gastric sleeve surgery reported in the literature is up to 120 min. Despite the patient's anatomical irregularities in this case, our surgery time was below the reported average; however, it is important to note that the surgeon's experience may have contributed to this outcome.

It has been demonstrated that LSG is a safe and effective procedure for patients with abnormal anatomies such as SIT, and it should be considered as a treatment option when other less invasive methods have failed, provided that the surgeon is capable of adapting the surgical techniques based on the specific clinical scenario.

## Conclusions

The LSG surgery has proven to be a safe and effective procedure for patients with SIT. Surgeons must be prepared for this rare condition and conduct a preoperative diagnosis to reduce complications. By modifying trocar positions and adapting surgical techniques, the procedure can be successfully performed, offering significant benefits to patients with SIT. Continued research and experience in managing these patients will further optimize outcomes in this unique population.

## References

[1] Amirbeigi A, Abbaslou F, Elyasinia F (2022). Laparoscopic sleeve gastrectomy in a patient with Situs Inversus Totalis: a case report and literature review. Ann Med Surg (Lond).

[2] Finkelstein EA, Trogdon JG, Cohen JW (2009). Annual medical spending attributable to obesity: payer-and service-specific estimates. Health Aff (Millwood).

[3] Fischer L, Hildebrandt C, Bruckner T (2012). Excessive weight loss after sleeve gastrectomy: a systematic review. Obes Surg.

[4] Catheline JM, Rosales C, Cohen R (2006). Laparoscopic sleeve gastrectomy for a super-super-obese patient with situs inversus totalis. Obes Surg.

[5] Atwez A, Keilani Z (2018). Laparoscopic Roux-en-Y gastric bypass in a patient with situs inversus totalis: case report, technical tips and review of the literature. Int J Surg Case Rep.

[6] Samaan M, Ratnasingham A, Pittathankal A (2008). Laparoscopic adjustable gastric banding for morbid obesity in a patient with situs inversus totalis. Obes Surg.

[7] Burvill A, Blackham R, Hamdorf J (2019). Laparoscopic sleeve gastrectomy in a patient with situs inversus totalis and Kartagener syndrome: an unusual surgical conundrum. BMJ Case Rep.

[8] Froylich D, Segal-Abramovich T, Pascal G (2018). Laparoscopic sleeve gastrectomy in patients with situs inversus. Obes Surg.

